# Thrombolytic Therapy for ST-Elevation Myocardial Infarction and High-Risk Pulmonary Embolism in a Non-percutaneous Coronary Intervention-Capable Hospital

**DOI:** 10.7759/cureus.93014

**Published:** 2025-09-23

**Authors:** Abdul-Subulr Yakubu, Hellen M Adok, Dzifa Ahadzi, Odalys Rivera, Prosper Akanbong

**Affiliations:** 1 Internal Medicine, Tamale Teaching Hospital, Tamale, GHA; 2 Pharmacy, Tamale Teaching Hospital, Tamale, GHA

**Keywords:** ghana, myocardial infarction, pulmonary embolism, reperfusion therapy, thrombolysis

## Abstract

Introduction

ST-elevation myocardial infarction (STEMI) and pulmonary embolism (PE) are common life-threatening emergencies encountered in clinical practice. Options for reperfusion therapy are often unavailable in resource-limited settings like Ghana. We present a review of our 19-month experience with systemic thrombolysis for patients with STEMI and hemodynamically unstable PE in Tamale Teaching Hospital (TTH) in Tamale, Ghana.

Methods

We retrospectively reviewed patients ≥ 18 years old with acute STEMI and PE managed in TTH from January 2023 to July 2024. STEMI was defined using standard criteria. Systemic thrombolysis was administered to eligible STEMI patients presenting within 12 hours of symptom onset. Hemodynamically unstable PE was defined as persistent hypotension (systolic blood pressure <90 mmHg for longer than 15 minutes), obstructive shock, or the need for cardiopulmonary resuscitation. We assessed treatment timelines, complications, and in-hospital outcomes.

Results

Of 42 patients with a mean age (± SD) of 56.6 ± 16.7 years with STEMI, 25 (59.5%) were male. Seven (16.7%) patients received systemic thrombolysis as the primary reperfusion strategy. For patients with STEMI, the median delay from symptom onset to first medical contact (FMC) was 10 hours (IQR 3.0-42.0), whilst the delay from FMC to thrombolysis was four hours (IQR 2-5 hours). Seventy-six patients with a mean age of 53.0 ± 18.1 years had confirmed pulmonary embolism, of which 35 (46.1%) were male. Systemic thrombolysis was administered to five hemodynamically unstable patients. Cost considerations largely determined the choice of thrombolytic agent used. Adverse events following thrombolysis included four cases of allergic reactions (all with streptokinase), two patients with minor bleeding involving skin puncture sites, and one patient with bleeding from the urethral meatus.

Conclusion

Reperfusion therapy for STEMI and PE remains challenging in resource-limited settings like Ghana. Systemic thrombolysis is feasible but is limited by systemic challenges, including the high cost of fibrin-specific thrombolytic agents and late presentation. Strategies to promote early presentation and timely thrombolysis, such as community education, access to timely electrocardiogram interpretation, and sustainable financing models, are needed.

## Introduction

The burden of cardiovascular disease (CVD) is increasing at an alarming rate across low- and middle-income countries (LMICs), where it has rapidly become a leading cause of morbidity and mortality [1]. This surge threatens to overwhelm fragile health systems, particularly in Africa, which are already strained by persistent infectious diseases [2]. Statistics reveal that a striking 80% of all cardiovascular deaths occur in LMICs, highlighting a profound global health disparity [3,4].

While the implementation of evidence-based guidelines has dramatically improved outcomes for CVD emergencies such as ST-elevation myocardial infarction (STEMI) in high-income countries, LMICs face substantial challenges in adopting these strategies, resulting in persistently suboptimal outcomes [5-7]. These challenges include frequent late patient presentation, significant diagnostic delays, and an inability to meet critical treatment timelines [8]. Furthermore, there is often limited availability of acute interventions, such as reperfusion therapy, and a profound lack of health financing schemes designed for emergency care [9].

In settings without access to advanced cardiac catheterization laboratories, systemic thrombolysis serves as an established, life-saving intervention for both STEMI and massive pulmonary embolism (PE) [10]. Data on the use and outcomes of thrombolytic therapy for STEMI and PE remain notably absent in many African countries, including Ghana. This study aimed to evaluate, for the first time in Ghana, the feasibility and outcomes of systemic thrombolysis for STEMI and hemodynamically unstable PE at Tamale Teaching Hospital (TTH) in Tamale, Ghana, specifically assessing treatment timelines, in-hospital complications, and mortality over a 19-month period. 

## Materials and methods

Study design and setting

This was a single-center, cross-sectional, retrospective observational study conducted in the Department of Internal Medicine of TTH between January 2023 and July 2024, involving adult patients with confirmed STEMI or PE. TTH is one of Ghana's five major public teaching hospitals. It has 800 beds and serves as the main referral hospital for the entire northern part of the country. The hospital's patient population is predominantly drawn from rural, agrarian communities with high levels of poverty, which present distinct challenges related to healthcare access, resource allocation, and the management of complex medical conditions.

As a facility without percutaneous intervention (PCI) capability, TTH lacked specialized cardiology services until late 2022, when the arrival of its first two trained cardiologists allowed the creation of a cardiology unit. This included a thrombolysis response team, made up of two cardiologists, a pharmacist, and a nursing team, that put into practice a locally adapted protocol for systemic thrombolysis.

Study population

One hundred and eighteen eligible participants were identified through the hospital’s electronic medical records. Most admissions originated in the emergency department, with the remainder being inpatient transfers. Patient care was managed by a team of emergency physicians, cardiologists, internists, and primary care physicians.

Inclusion and Exclusion Criteria

Eligible participants were aged 18 years or older with a diagnosis of STEMI or computed tomography pulmonary angiography (CTPA)-confirmed PE. STEMI was defined by standard electrocardiographic (ECG) criteria, in accordance with the Fourth Universal Definition of Myocardial Infarction (new ST-elevation at the J-point in two contiguous leads ≥ 1 mm except in leads V2 and V3, where the cut-off was ≥ 2 mm in men 40 years and older, ≥ 2.5 mm in men under 40 years, or ≥ 1.5 mm in women, regardless of age) [11]. Eligible candidates for systemic thrombolysis included STEMI patients presenting within 12 hours of symptom onset, as well as patients with high-risk PE characterized by persistent hypotension (systolic blood pressure <90 mmHg for more than 15 minutes), obstructive shock, or the need for cardiopulmonary resuscitation. Contraindications to thrombolytic therapy were systematically evaluated using the hospital's standardized thrombolysis checklist (Appendix A). Patients were excluded if elevated cardiac troponins or myocardial injury were attributable to conditions other than acute ischemia (such as renal failure, chronic heart failure, or myocarditis). Those who were discharged against medical advice were also excluded.

Thrombolysis Protocol

Tenecteplase was administered as a single bolus injection over 10 seconds based on the patient's body weight. Streptokinase was administered as a continuous infusion of 1.5 million units over 60 minutes for STEMI and over 120 minutes for PE. Alteplase was administered at a dose of 100 mg infused over two hours for PE. 

Data collection

Trained research assistants, supervised by a cardiologist, extracted data from the hospital's electronic medical records using a data collection template. Data collected included demographics, risk factors, symptoms, treatment delays, therapies received (including thrombolytic agents), and in-hospital outcomes. All abstractors were trained with explicit definitions for all variables. Every single chart was independently abstracted by two trained team members whose entries were systematically compared by a third senior researcher (a cardiologist). All identified discrepancies were flagged and resolved through consensus review by the principal investigators. A cardiologist reviewed ECGs and echocardiograms to confirm diagnoses and identify complications. Laboratory results, including troponin levels, were analyzed to confirm diagnoses and assess comorbidities. We assessed treatment timelines, including door-to-needle time (time between a patient's arrival and the administration of the thrombolytic agent) and symptom-to-thrombolysis intervals, procedure-related complications, and mortality as primary endpoints. Length of hospital stay was assessed as a secondary endpoint.

Statistical analysis

All statistical analyses were performed using IBM SPSS Statistics software, version 21 (IBM Corp., Armonk, NY). Categorical variables were expressed as counts and percentages, while continuous variables were presented as means ± standard deviations (if normally distributed) or medians with interquartile ranges (if non-normally distributed). The results were summarized into tables.

Ethical considerations

The study protocol received ethical approval from the Ethics Committee of TTH (TTHERC/05/07/23/02). This study was conducted in accordance with local guidelines and regulations and the ethical principles of the Declaration of Helsinki.

## Results

A total of 118 patients, comprising 42 patients with STEMI and 76 with confirmed PE, were analyzed. Table 1 summarizes the baseline characteristics and cardiovascular risk factors of the participants. For patients with STEMI, dyspnea (90.5%) and chest pain (88.1%) were the most common presenting symptoms. Gastrointestinal symptoms such as nausea, vomiting, and abdominal pain were present in 14.3%, whilst two patients (4.8%) presented with syncope. 10 (23.8%) suffered a cardiac arrest at presentation. Among patients with PE, dyspnea (94.7%), chest pain (61.8%), easy fatigue (59.2%), and syncope (7.9%) were among the commonest presenting symptoms. The most common cardiovascular risk factor was hypertension. 

**Table 1:** Demographics and baseline characteristics of the participants IQR: interquartile range; PE: pulmonary embolism; SD: standard deviation; STEMI: ST-elevation myocardial infarction

Variable	n%
Sociodemographic information	
Age in years (mean ± SD), N=118	54.9 ± 17.6
STEMI (mean ± SD), N=42	56.6 ±16.7
PE (mean ± SD), N=76	53.0 ± 18.1
Gender (males), N=118	60 (50.8%)
STEMI (males), N=42	25 (59.5%)
PE (males), N=76	35 (46.1%)
Health insurance coverage, N=118	99 (83.9%)
Cardiovascular risk factors (STEMI), N=42	
Hypertension	32 (76.2%)
Diabetes mellitus	11 (26.2%)
History of myocardial infarction	6 (14.3%)
History of stroke	5 (11.9%)
Dyslipidemia	3 (7.1%)
Chronic kidney disease	4 (9.5%)
Obesity	1 (2.4%)
Cardiovascular risk factors (PE), N=76	
Hypertension	41 (53.9%)
Diabetes mellitus	13 (17.1%)
History of PE	9 (11.8%)
History of deep vein thrombosis	16 (21.1%)
History of stroke	5 (6.6%)
Dyslipidemia	1 (1.3%)
Chronic kidney disease	5 (6.6%)
Obesity	11 (14.5%)
Smoking	1 (1.3%)
Presenting symptoms, N=118	
Dyspnea	110 (93.2%)
Chest pain	84 (71.2%)
Easy fatigability	79 (66.9%)
Gastrointestinal complaints	12 (10.2%)
Syncope	8 (6.8%)
Cardiac arrest	10 (8.5%)
Duration of hospital stay in days (median, IQR), N=118	8.0 (4.0-11.0)
Systolic blood pressure in mmHg (mean ± SD), N=118	126.9 ± 30.3
Diastolic blood pressure in mmHg (mean ± SD), N=118	82.2 ± 18.5
Heart rate in beats per minute (mean ± SD), N=118	96 ± 21

Seven (16.7%) patients received systemic thrombolysis as the primary reperfusion strategy for STEMI. Streptokinase was the most common thrombolytic agent administered. For patients presenting with STEMI, the median delay from symptom onset to first medical contact (FMC) was 10 hours (IQR 3.0-42.0), whilst the delay from FMC to thrombolysis was 4 hours (IQR 2.0-5.0 hours). Common complications in patients with STEMI included heart failure (35.7%), cardiac arrhythmias (33.3%), asymptomatic left ventricular systolic dysfunction (11.9%), and refractory angina (7.1%). Two patients developed ventricular septal defects, and one had a left ventricular aneurysm. Systemic thrombolysis was administered to five hemodynamically unstable patients with PE. Right heart failure was documented in 18 (23.7%) patients managed for PE.

Table 2 summarizes the thrombolytic agents administered for each clinical indication, with cost considerations being the predominant factor influencing agent selection. Allergic reactions were observed in four patients, all in the streptokinase group. Minor bleeding events occurred in three patients, manifesting as bleeding from skin puncture sites in two patients and the urethral meatus in one patient. 

**Table 2:** Summary of thrombolytic agents administered PE: pulmonary embolism; STEMI: ST-elevation myocardial infarction

Indication	Thrombolytic agent			
	Streptokinase	Alteplase	Tenecteplase	Total
STEMI	5	0	2	7
PE	3	1	1	5
Total	8	1	4	12

Out of 42 patients with STEMI, 10 (23.8%) died during admission. One out of seven patients who received thrombolysis died (14.3%), whilst nine out of 35 patients (25.7%) who did not receive thrombolysis died. 28 (36.8%) in-hospital deaths were recorded among patients with PE. Table 3 presents the in-hospital mortality outcomes for patients diagnosed with STEMI and PE. 

**Table 3:** In-hospital mortality of patients with STEMI and PE PE: pulmonary embolism; STEMI: ST-elevation myocardial infarction

Diagnosis			Thrombolysis administered		Total
			Yes	No	
STEMI	Deaths	Yes	1	9	10
		No	6	26	32
	Total		7	35	42
PE	Deaths	Yes	3	25	28
		No	2	46	48
	Total		5	71	76
Total	Deaths	Yes	4	34	38
		No	8	72	81
	Total		12	106	118

## Discussion

Systemic thrombolysis is an established life-saving intervention for both STEMI and massive PE [10,12]. This study represents, to our knowledge, the first report on thrombolytic therapy for STEMI and PE in Ghana, underscoring both the profound underutilization of this reperfusion strategy and the practical challenges associated with its delivery.

Primary PCI for STEMI offers more complete coronary flow restoration and a significantly lower risk of intracranial hemorrhage compared to thrombolysis [12,13]. However, in resource-poor countries where PCI capabilities are scarce or inaccessible, thrombolytic therapy continues to serve as the predominant reperfusion strategy [14,15]. Despite this, thrombolysis uptake in LMICs remains suboptimal due to multifaceted barriers, including infrastructure limitations, financial constraints, and systemic healthcare challenges [16]. The observed in-hospital mortality rate of 23.8% among STEMI patients aligns with the elevated mortality rates reported throughout Africa, underscoring the ongoing challenges in acute coronary syndrome (ACS) management across the continent [17].

Clinical evidence demonstrates that even though thrombolysis for STEMI maintains benefit when administered within six to 12 hours of symptom onset, maximal efficacy is achieved when treatment is initiated within the critical first 2-hour window [12]. In this study, the median delays were 10 hours from symptom onset to FMC and 4 hours from FMC to thrombolysis, consistent with studies highlighting late presentation as a hallmark of acute ACS in many sub-Saharan African countries [7,15,18,19]. Contributing factors include lack of patient awareness, inadequate emergency ambulance services, low clinical suspicion among physicians, and delays in obtaining ECGs [19,20]. This challenge is further compounded by the fact that patients must pay out of pocket for thrombolytic agents, a cost that exceeds the financial means of most. Minimizing treatment delays requires a multi-faceted approach, including public awareness campaigns, training in early ECG interpretation, efficient ambulance services, and sustainable financing. The inaccessibility of thrombolytic agents for most participants in this study, 83.9% of whom had public insurance, is due to their exclusion from Ghana's national health insurance coverage. Current guidelines recommend that STEMI patients arriving at a non-PCI-capable hospital should receive prompt fibrinolysis unless timely transfer for primary PCI (within ≤120 minutes of FMC) is feasible [21].

The diagnosis of PE remains particularly challenging in resource-constrained settings. Thrombolytic therapy is a viable option in patients with hemodynamically unstable PE without absolute contraindications, as their potential benefits outweigh the risk of a life-threatening bleed [22]. Despite this, there remains a wide variation in thrombolytic use for PE, and hospitals vary widely in thrombolysis rates for hemodynamically unstable PE [23,24]. In sub-Saharan Africa, there is limited data on the utilization of thrombolysis in patients with PE. Due to resource limitations and delays, only a small proportion of patients with high-risk PE receive thrombolytic therapy, despite evidence that it is highly effective and can significantly reduce in-hospital mortality in these settings [25]. The observed high in-hospital mortality rate and frequent occurrence of right heart failure among confirmed PE cases in this study emphasize the need for improved early diagnostic capabilities, more accurate risk stratification, and timely initiation of appropriate therapy.

Thrombolysis, however, carries inherent limitations: reperfusion fails in approximately 50% of cases, while contraindications further exclude some otherwise eligible patients [26]. These constraints underscore that even optimized thrombolysis programs cannot fully address the reperfusion needs of all patient populations, necessitating parallel efforts to develop transcatheter capabilities while improving thrombolytic access. As the burden of cardiovascular diseases continues to increase in LMICs, more emphasis should be placed on primary prevention and better control of their risk factors, such as hypertension and diabetes mellitus, whilst improving the capacity to handle complications in a context-appropriate manner [27].

This study has several important limitations, including its relatively small sample size, single-center design, and absence of a comparator group. The retrospective observational design restricts causal inference, and the relatively small sample size limits the statistical power and generalizability of the findings. The lack of long-term follow-up confines conclusions to in-hospital outcomes only. Nevertheless, this study demonstrates the feasibility of implementing a thrombolysis protocol in resource-constrained settings, despite significant systemic challenges.

## Conclusions

This study demonstrates that while systemic thrombolysis is a feasible reperfusion strategy for STEMI and high-risk PE in resource-limited settings like northern Ghana, substantial systemic challenges persist. There is an urgent need for community education programs to reduce care-seeking delays, sustainable financing models for thrombolytic agents, and protocol optimization in non-PCI-capable centers. To overcome its limited capacity for cardiovascular emergency management, Ghana must adopt context-appropriate acute care solutions while strengthening primary prevention and risk factor control as a core public health strategy.

## Figures and Tables

**Figure 1:**
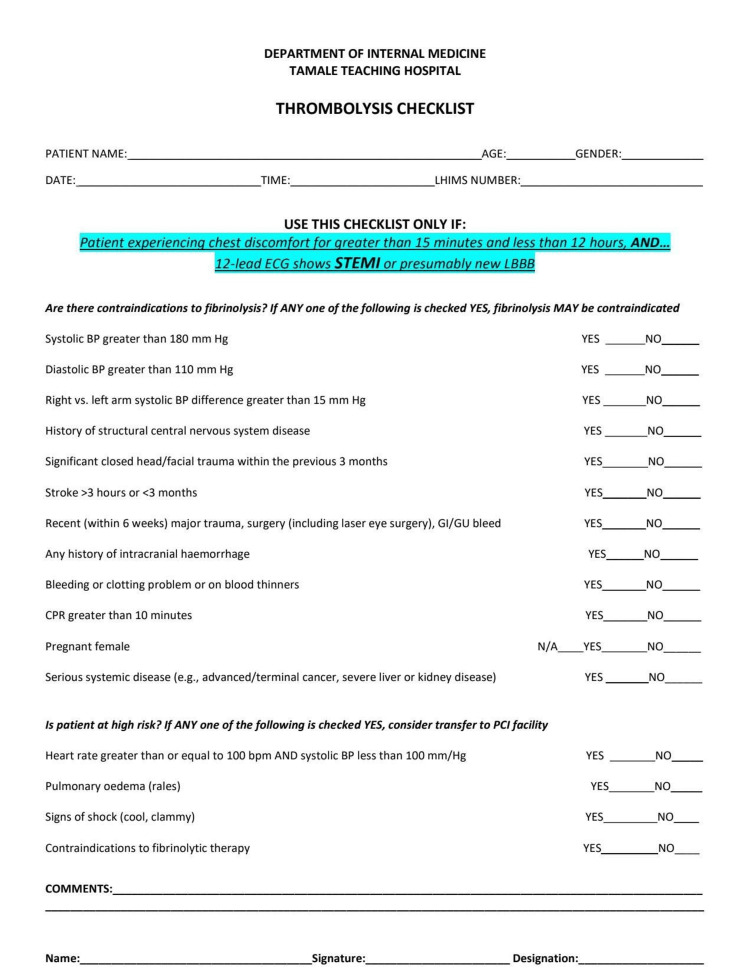
Thrombolysis checklist for Tamale Teaching Hospital BP: blood pressure; CPR: cardiopulmonary resuscitation; GI: gastrointestinal; GU: genitourinary; LBBB: left bundle branch block; PCI: percutaneous coronary intervention; STEMI: ST-elevation myocardial infarction

## Appendices

Appendix A 
